# Fatal Pediatric Motor Vehicle Crashes on U.S. Native American Indian Lands Compared to Adjacent Non-Indian Lands: Restraint Use and Injury by Driver, Vehicle, Roadway and Crash Characteristics

**DOI:** 10.3390/ijerph14111287

**Published:** 2017-10-25

**Authors:** Shin Ah Oh, Chang Liu, Joyce C. Pressley

**Affiliations:** 1 Department of Biostatistics, Columbia University, 722 West 168th Street, New York, NY 10032, USA; so2461@cumc.columbia.edu; 2 Department of Epidemiology, Columbia University; 722 West 168th Street, New York, NY 10032, USA; cl3321@cumc.columbia.edu; 3 Department of Health Policy and Management, Columbia University, 722 West 168th Street, New York, NY 10032, USA; 4 Center for Injury Epidemiology and Prevention, Columbia University, 722 West 168th Street, New York, NY 10032, USA

**Keywords:** motor vehicle crash, Indian lands, restraint use

## Abstract

There are large disparities in American Indian pediatric motor vehicle (MV) mortality with reports that several factors may contribute. The Fatality Analysis Reporting System for 2000–2014 was used to examine restraint use for occupants aged 0–19 years involved in fatal MV crashes on Indian lands (*n* = 1667) and non-Indian lands in adjacent states (*n* = 126,080). SAS GLIMMIX logistic regression with random effects was used to generate odds ratios (OR) with 95% confidence intervals (CI). Restraint use increased in both areas over the study period with restraint use on Indian lands being just over half that of non-Indian lands for drivers (36.8% vs. 67.8%, *p* < 0.0001) and for pediatric passengers (33.1% vs. 59.3%, *p* < 0.0001). Driver restraint was the strongest predictor of passenger restraint on both Indian and non-Indian lands exerting a stronger effect in ages 13–19 than in 0–12 year olds. Valid licensed driver was a significant predictor of restraint use in ages 0–12 years. Passengers in non-cars (SUVs, vans and pickup trucks) were less likely to be restrained. Restraint use improved over the study period in both areas, but disparities failed to narrow as restraint use remains lower and driver, vehicle and crash risk factors higher for MV mortality on Indian lands.

## 1. Introduction

Motor vehicle (MV) crashes are the leading cause of unintentional injury in the U.S. for ages 0 to 19 years [1]. Racial and ethnic disparities exist in MV-related mortality, with American Indians aged 0 to 19 years having higher mortality [2]. Historical trends have generally shown maintenance or widening of these disparities despite a lowering of the actual mortality rate in all race and ethnic groups [3].

This occurred as MV mortality rates fell in all race and ethnic groups, but with smaller improvements for American Indian/Alaskan Natives that produced an actual widening of MV occupant disparities [3]. Appropriate restraining of children in motor vehicles is effective in lowering child MV crash mortality [4,5,6], but American Indian pediatric passengers are reported to be less likely to be properly restrained [3,7,8,9,10], thus potentially increasing the risk of mortality. Several risk factors for increased risk of injury and mortality among American Indians have been identified [11,12,13,14,15], although there are also reports of racial misclassification for American Indians [16,17,18]. In this study, we avoid some of the potential bias that could be introduced by racial misclassification of American Indians by examining whether the crash occurred on federally-designated Indian lands or nearby lands that were not designated as such. Indian lands have a higher percentage of single-vehicle fatal crashes that have been associated with increased likelihood of fatality [15]. Within those geographic categories, we report race and ethnicity, although our multivariable models do not depend on racial classifications.

This study examines fatal motor vehicle crashes involving transport of a pediatric passenger in states with federally recognized Indian lands and compares crashes occurring on Indian lands to those occurring on non-Indian lands in the states that contain these Indian lands. In particular, among a pediatric population aged 0–19 years involved in a fatal MV crash, we examine: (1) predictors of restraint use on Indian lands compared to non-Indian lands by age, driver and vehicle characteristics, and (2) crash, environmental and roadway characteristics on Indian lands and non-Indian lands.

## 2. Materials and Methods

### 2.1. Data Source

The data used in this study is from the Fatality Analysis Reporting System (FARS) (2000–2014) publicly released by National Highway Traffic Safety Administration (NHTSA) [19]. FARS is a nationwide census of fatal injuries in MV traffic crashes on U.S. public roads and contains variables that characterize person, vehicle and crash factors.

### 2.2. Study Population

**Figure 1:**
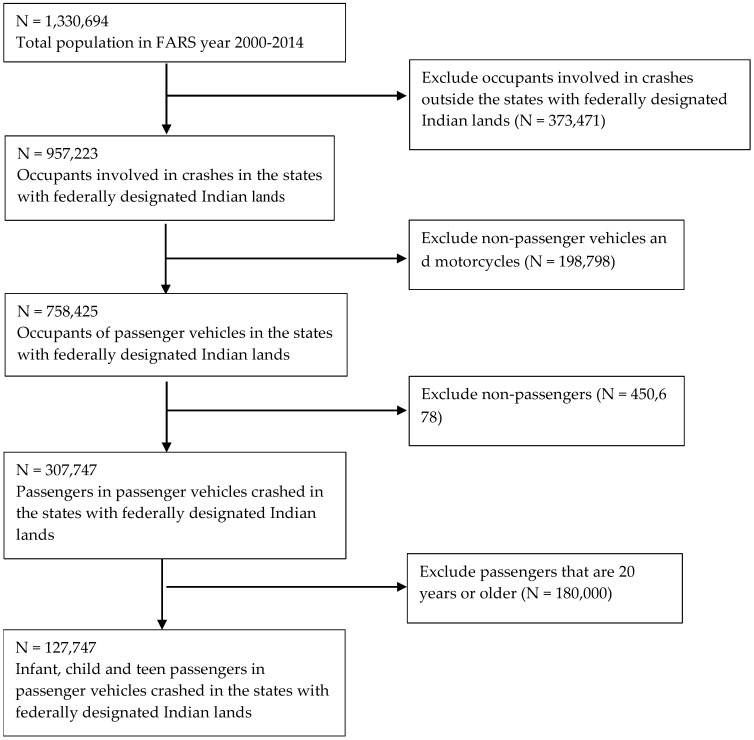
Population flow diagram of the study population of passengers aged 0–19 years, FARS 2000–2014.

The study was limited to the following U.S. states that contain federally designated Indian lands: Alabama, Arizona, California, Colorado, Connecticut, Florida, Idaho, Indiana, Iowa, Kansas, Louisiana, Maine, Massachusetts, Michigan, Minnesota, Mississippi, Montana, Nebraska, Nevada, New Mexico, New York, North Carolina, North Dakota, Oklahoma, Oregon, Rhode Island, South Carolina, South Dakota, Texas, Utah, Virginia, Washington, Wisconsin, and Wyoming [20]. Of the 957,223 crashes in the states with federally designated Indian lands, 127,747 (13.3%) crashes were to passenger vehicles transporting an infant, child or teen passenger (Figure 1).

### 2.3. Variable Classification

#### 2.3.1. Person-Level Characteristics

- Passenger Restraint Status (Outcome). This is a dichotomous variable, restrained or not restrained, with use of any type of restraint being categorized as restrained. Improper restraint use or inappropriate restraint use for the age of the child were not examined outside of the restraint category.
- Passenger Mortality. This was categorized dichotomously from injury severity data in FARS. If the passenger was known to have a fatal injury the passenger was categorized as having died.
- Passenger Age and Gender. Passenger age was categorized as follows: 0–2, 3–8, 9–12, 13–14, 15–17, and 18–19 years. Gender was characterized as male or female.
- Passenger Seating Position. This was comprised of 5 categories—front, rear right or left side, rear middle, other or unknown. Passengers travelling in the sleeper section of the vehicle, cargo area or beyond fifth row of seats were categorized as ‘other’.
- Driver Age and Gender. Driver age was categorized for the 76,428 drivers as follows: less than 20 years old, 20–44, 45–64, and 65 years or older. Gender was characterized as male or female.
- Driver License Validity. License was considered valid at the time of the crash if the driver had a valid learner’s permit, intermediate or full license or a temporary license. A driver’s license was considered invalid if it had been suspended, revoked, expired, or cancelled at the time of crash.
- Driver Drug or Alcohol Status. This variable was categorized as negative, positive or not tested. A positive status included any of the following: (1) Police reported alcohol involvement; (2) driver blood alcohol concentration greater than or equal to 0.01; or (3) the driver otherwise had a positive drug or alcohol test result. If the driver didn’t match any of these criteria and had no missing data in any of these variables, then the driver was categorized as negative. If the records from all those variables indicate that the driver was not tested, then the driver was categorized as not tested and alcohol and drug status as unknown/missing.
- Driver Previous Moving Violation. This was a dichotomous variable of yes or no based on having received a citation within 3 years of the crash date for previously driving while intoxicated, speeding or another moving violation.
- Driver Race and Ethnicity in Death Certificate. Driver race was categorized as White, non-Hispanic; Black, non-Hispanic, Hispanic, White or Black; Native American; Asian or Pacific Islander; and Multiple/other. Race and ethnicity were available only for occupants who died.

#### 2.3.2. Vehicle-Level Characteristics

- Vehicle Model Year. Vehicle model year was examined as a categorical variable (<1994, 1994–1997, 1998–2004, 2005–2008, 2009–2011, 2012–2014) [21].
- Vehicle Model Type. Vehicle type was categorized as passenger car, utility vehicle (SUV), van or pickup truck. Vehicles such as large trucks, motorcycles and buses were excluded.

#### 2.3.3. Crash-Level Characteristics

- Day/night. Day was defined as 6:00 a.m. to 5:59 p.m. and night was defined as 6:00 p.m. to 5:59 a.m.
- Weekday/weekend. Social weekend was defined as Friday at 5:00 p.m. to Sunday at 4:59 p.m., and times outside this were categorized as weekday [22].
- Rollover or Ejection. If the vehicle and the occupants experienced either rollover or ejection or both, the data was categorized as having had rollover or ejection.
- Manner of Collision. Manner of collision was analyzed by categorizing the collision types as rear-end, head-on, angle, sideswipe or other. Non-collision was used as a category for the crashes that did not involve collision with motor vehicle in transport.

#### 2.3.4. Road Characteristics

- Number of Lanes. Number of lanes was categorized as one, two, three, four, five and six or more.
- Trafficway. The categories for this variable were one-way; two-way, divided; two-way, not divided; and other. Both unprotected median and positive median barriers were considered divided.
- Route Signing. This variable was categorized in FARS as interstate, highway, country road, local street or other.
- Traffic Devices and Signs. This was categorized as no controls, traffic signals, regulatory signs or other.
- Traffic Control Device Functioning. Three categories were used—not functioning properly, functioning properly and no controls.

### 2.4. Statistical Analysis

Chi-square tests were used to assess the associations between the geographic area (Indian land vs. non-Indian land) status of crash location and potential covariates. Significance was defined as having a *p*-value of less than or equal to 0.05. PROC GLIMMIX in SAS with random effects was used to generate the odds ratios and 95% confidence intervals for Indian lands and non-Indian lands, to account for the effects of clustering by multiple passengers in the same vehicle. Separate models were constructed for Indian lands and non-Indian lands to examine difference in predictors of restraint use on each category of lands. The population was further stratified by age—0 to 12 years and 13 to 19 years—as seating position recommendations/guidelines exist and vary by age [23,24]. Variables such as roadway characteristics with large quantities of missing data are reported but not included in the multivariable models. Race and ethnicity were not included in the multivariable models because the variable was only reported for drivers who died. All analyses were conducted using SAS 9.4 (SAS Institute, Inc., Cary, NC, USA) [25].

## 3. Results

The study population consisted of 127,747 passengers aged 0 to 19 years being driven in 76,428 passenger vehicles. Of those, 126,080 (98.7%) crashed outside federally designated Indian lands and 1667 (1.3%) on Indian lands.

**Figure 2:**
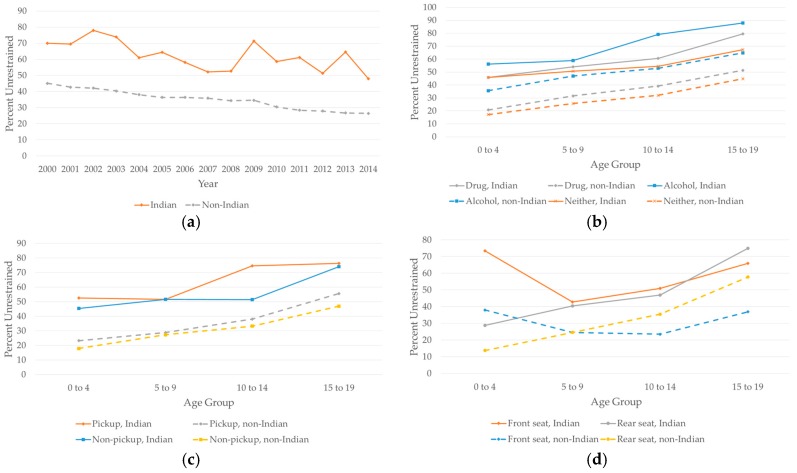
Percentage of unrestrained passengers aged 0–19 years old by different factors on Indian lands compared to non-Indian lands: (**a**) Percentage of unrestrained passengers by year; (**b**) Percentage of unrestrained passengers for driver drug and alcohol use; (**c**) Percentage of unrestrained passengers for pickup trucks and non-pickup trucks; and (**d**) Percentage of unrestrained passengers for front seat and rear seat.

Restraint use increased from 2000 to 2014 on both Indian and non-Indian lands, with passengers and drivers on Indian lands consistently showing lower restraint use (Figure 2a).

Among those with known restraint status, 36.8% of the drivers on Indian lands were restrained, compared to 67.8% on non-Indian lands (*p* < 0.0001). One third of (33.1%) of the passengers on Indian lands were restrained, compared to 59.3% on non-Indian lands (*p* < 0.0001). Passenger restraint status depended more on whether the vehicle was on Indian lands at the time of the crash and less on other covariates (Figure 2b,c). Drivers who were positive for drugs or alcohol were more likely to have unrestrained passengers on both Indian and non-Indian lands (Figure 2b), and passengers of pickup trucks for both Indian lands and non-Indian lands were less likely to be restrained (Figure 2c). Young passengers on Indian lands were less likely to conform to NHTSA rear-seating guidelines (Figure 2d).

### 3.1. Driver Characteristics

Drivers of vehicles crashing on Indian lands differed from those of non-Indian land drivers by age, gender, belt status, injury severity, license validity, race and ethnicity and previous moving violations (Table 1). The majority of drivers who crashed and died on Indian lands compared to non-Indian lands were reported to be Native American (64.7% vs. 2.0%, *p* < 0.0001). Driver mortality was higher on Indian lands compared to non-Indian lands (34.2% vs. 29.2%, *p* < 0.0001) as was passenger injury (60.4% vs. 55.0%, *p* < 0.0001) (Table 1).

Drivers crashing on Indian lands were more than twice as likely to have an invalid license compared to those on non-Indian lands (35.7% vs. 15.8%, *p* < 0.0001). Of the 39.2% of drivers on Indian lands who were tested for drugs or alcohol, none tested negative (*p* < 0.0001). Drivers of vehicles crashing on Indian lands were less likely to be restrained and were more likely to die as a result of the crash.

### 3.2. Passenger Characteristics

There were no significant differences in the age of passengers between Indian lands and non-Indian lands. Passengers were less likely to be restrained on Indian lands and were more likely to be injured than on non-Indian lands. Passengers of crashes on Indian lands were more likely to be seated in areas of the vehicle other than usual passenger seats (Table 1).

In the multivariable adjusted model, passengers on Indian lands were less likely to be restrained on Indian lands than on non-Indian lands (Table 2). The effect of Indian land crash site on likelihood of being restrained is more pronounced for passengers aged 0–12 (OR = 0.535, 95% CI 0.416, 0.715) than for passengers aged 13–19 years (OR = 0.635, 95% CI 0.500, 0.807), controlling for driver and vehicle characteristics.

#### 3.2.1. Passenger Restraint Use for Indian Lands

**Figure 3:**
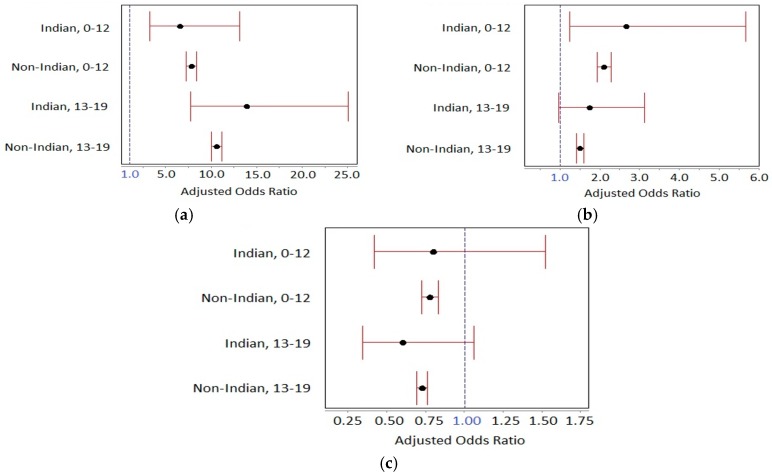
Independent predictors of passenger (aged 0–19 years) restraint use for Indian lands and non-Indian lands by age group, FARS 2000–2014: (**a**) Driver restraint use as an independent predictor of passenger (aged 0–19 years) restraint use (Adjusted for driver age, gender, license validity, drug/alcohol status and vehicle model type); (**b**) Driver valid license as an independent predictor of passenger (aged 0–19 years) restraint use (Adjusted for driver age, gender, restraint status, drug/alcohol status and vehicle model type); (**c**) Vehicle type as an independent predictor of passenger (aged 0–19 years) restraint use (Adjusted for driver age, gender, restraint status, license validity and drug/alcohol status).

In the multivariable adjusted model, passengers across all ages from 0 to 19 years were more likely to be restrained when the driver was restrained (Table 3), controlling for driver age, gender, license validity, drug or alcohol test results and vehicle model type. The odds of passengers being restrained when the driver was restrained is greater for passengers aged 13–19 compared to passengers aged 0–12 years (Figure 3a). Passengers aged 0–12 were more likely to be restrained when the driver had a valid license, but this effect was not significant for passengers aged 13–19 years (Figure 3b). Passengers aged 0–12 years were more likely to be belted with drivers aged 20–44, and passengers aged 13–19 were more likely to be belted with drivers aged 45–64 years.

Passengers were generally less likely to be restrained in vehicles other than passenger cars (SUVs, vans, and pickups) (Figure 3c). Driver gender was not a significant predictor of passenger belt status in either unadjusted and adjusted models; a positive drug or alcohol test result was significant in unadjusted models but no longer significant in adjusted models.

#### 3.2.2. Passenger Restraint Use for Non-Indian Lands

Passengers across all ages from 0 to 19 years were more likely to be restrained when the driver was restrained and had a valid license, but with smaller odds ratios compared to Indian lands, controlling for driver age, gender, license validity, drug or alcohol test results and vehicle model type (Table 4). Again, the odds of passengers being restrained when the driver was restrained was greater for passengers aged 13–19 compared to passengers aged 0–12 years (Figure 3a). Driver gender was a significant predictor for both unadjusted and adjusted models in passengers aged 0–12 years, but not for adjusted models of teen passengers. Drug or alcohol test results were significant only in unadjusted models, and only between those not tested and those who tested negative. Passengers were less likely to be belted in vehicles other than passenger cars (SUVs, vans, and pickups) (Figure 3c). Unadjusted comparisons between vans and passenger cars for passenger restraint status show a reversal of effect when adjusted for other variables.

### 3.3. Vehicle Characteristics

Vehicle model year and model type were associated with Indian land status of the crash site. Indian lands had a higher proportion of pre-1994 vehicles than non-Indian lands (*p* < 0.0001), and a higher proportion of pickup trucks than non-Indian lands (*p* < 0.0001) (Table 1).

### 3.4. Crash Characteristics

Crash time (day/night) and day (weekday/weekend), as well as rollover, ejection and manner of collision were all significantly associated with Indian land status. Vehicles that crashed on Indian lands were twice as likely to experience rollover (54.1% vs. 27.1%, *p* < 0.0001), and drivers of vehicles on Indian lands were twice as likely to be ejected upon collision (22.9% vs. 11.1%, *p* < 0.0001) (Table 1).

Single motor vehicle crashes consisted of 63.4% of all crashes on Indian lands, compared to 44.5% on non-Indian lands. Crashes in angle with other motor vehicles consisted of 30.1% of all crashes on non-Indian lands, compared to 13.9% on Indian lands (*p* < 0.0001) (Table 1).

### 3.5. Roadway Characteristics

Number of lanes, trafficway (one/two-way), traffic devices and signs and the functioning status of traffic control devices around the crash site had more than 70% of the data missing for both Indian lands and non-Indian lands. Analysis of reported data showed that crashes occurred mostly on highways (50.3%) and country roads (21.8%) for those on Indian lands, and on highways (40.6%) and local streets (20.1%) for those on non-Indian lands (Table 1).

## 4. Discussion

In many countries that also contain native population jurisdictions such as is found in the U.S., particular consideration should be given to examination and ongoing monitoring of the health impacts in geographic areas where motor vehicle laws and regulations differ from best practices. Examining these areas separately and providing the findings to local entities can stimulate local culturally-sensitive engagement in setting intervention priorities and in designing culturally appropriate solutions and interventions, including crafting of laws or regulations, aimed at lowering the burden of motor vehicle injury in the vulnerable populations that tend to live in these areas.

The strongest predictor of a pediatric passenger being unrestrained was whether the crash occurred on Indian lands compared to non-Indian lands in states containing these lands. Passenger restraint depended more on whether the vehicle crashed on Indian lands than on other covariates. This finding was surprisingly more pronounced in teens than in younger passengers aged 0–12 years.

There were several significant differences in the characteristics of crashes on Indian lands compared to non-Indian lands. Within geographic regions, driver restraint was a highly important factor, a finding consistent with what is generally known [9,11,15]. Environmental characteristics appear to play an important role with crashes on Indian lands being more likely to be on two lane roads and to be single vehicle collisions. There was one difference in particular that may have contributed to smaller disparities in mortality on Indian lands than would have been expected given the vast number of characteristics present in higher proportions on Indian lands that are known to contribute to higher mortality. In particular, the higher proportion of pickup trucks to passenger cars is generally associated with heavier vehicles and vehicles in which passengers sit higher and may be more protected than in those in passenger cars. This study did not address how these findings compare when controlling for rural and sparsely populated lands, a factor that should be addressed in future study [26]. The relative importance of some key covariates varied across passenger age categories that deserve further examination.

Our findings are consistent with earlier studies that showed American Indian/Alaskan Native (AI/AN) passengers aged 0–12 years of age are less likely to be in compliance with NHTSA recommended rear seating guidelines [27]. Our study extends these findings by demonstrating continued disparities in the noncompliance with guidelines across geographic jurisdictions. This issue is especially alarming, given that a crash on Indian lands is more predictive of being unrestrained for passengers aged 0–12 than for those aged 13–19 years.

Driver belt status was a strong predictor of passenger restraint use for teens on Indian lands where passengers of restrained drivers had overwhelming odds of being restrained compared to those travelling with unrestrained drivers. There was a much larger proportion of drivers unrestrained on Indian lands, and teen passenger restraint status mirrored that of the drivers transporting them [28]. Passengers aged 0–12 years on Indian lands being transported by drivers with valid driver licenses had the highest odds of being restrained. Our finding that the percentage of unrestrained passengers is the highest with drivers who tested positive for alcohol, followed by those tested positive for drugs and those tested positive for neither or who were not tested is consistent with previous studies [12,13].

Rollovers and ejections, previously reported to be associated with higher mortality, were significantly higher on Indian lands, which could have contributed to higher passenger mortality and injury, especially in those who were unrestrained. Furthermore, single vehicle collisions occurred more frequently on Indian lands and were more likely to result in rollover of vehicles than other types of collisions.

This study has limitations. Although this study was limited to the crashes in states with federally recognized Indian lands, there is significant heterogeneity within the Indian land category. We did not have variables in our data set to facilitate investigation of these differences. Our study only investigated passenger restraint status as restrained or unrestrained and did not consider the importance of child restraint types across younger age groups. We did not take holidays into account because holidays vary significantly across cultures, religions and geographic areas within the U.S. and across the geographic areas under study. There are variations in strength of laws and the enforcement of these laws within each Indian nation, as well as social structures that could play a contributing role. Of significance, is the disparities in the proportions of missing data in important variables. We had limited data on travel speed for both jurisdictions that precluded our investigation of this important factor. The issue of missing data was more pronounced for roadway characteristics variables, for which some of the percentages of missing data were as high as three quarters of the study population. Furthermore, percentages of missing data for most variables were consistently higher for crashes on Indian lands than for crashes on non-Indian lands. This suggests the need for improvement in data collection in both jurisdictions but particularly on Indian lands.

## 5. Conclusions

These findings suggest that there are marked differences in crash characteristics between the Indian lands and non-Indian lands with regard to vehicle, passenger, driver and crash characteristics that have been linked to higher MV mortality and morbidity. Infant, child and teen restraint patterns continue to be significantly lower on Indian lands compared to adjacent non-Indian lands. Although restraint use improved over the study period on both Indian and non-Indian lands, disparities remained and failed to narrow. Disparities in data quality between Indian lands and non-Indian lands were an obstacle to assessing several key risk factors. This pattern, in conjunction with differing environmental characteristics, is associated with greater pediatric endangerment on Indian lands compared to non-Indian lands.

## Figures and Tables

**Table 1:** Characteristics of crashes on federally recognized Indian lands and non-Indian lands with an infant, child and teen passenger aged 0 to 19 years, FARS 2000–2014.

Variables	Indian Lands, *n* (%) *	Non-Indian Lands, *n* (%) *	Total *	Chi-Square (*p*-Value)
**Total**	1667 (1.30)	126,080 (98.70)	127,747	
**Driver Characteristics**	868 (1.14)	75,359 (98.86)	76,227	
Driver age (years)				182.2 (<0.0001)
<20	255 (29.38)	22,242 (29.51)	22,497 (29.51)	
20 to 44	490 (56.45)	41,523 (55.10)	42,013 (55.12)	
45 to 64	87 (10.02)	9607 (12.75)	9694 (12.72)	
≥65	11 (1.27)	1760 (2.34)	1771 (2.32)	
Unknown	25 (2.88)	227 (0.30)	252 (0.33)	
Driver gender				85.8 (<0.0001)
Male	502 (57.84)	44,716 (59.34)	45,218 (59.32)	
Female	354 (40.78)	30,537 (40.52)	30,891 (40.53)	
Unknown	12 (1.38)	106 (0.14)	118 (0.15)	
Driver belt status				392.7 (<0.0001)
Restrained	319 (36.75)	51,065 (67.76)	51,384 (67.41)	
Unrestrained	416 (47.93)	19,642 (26.06)	20,058 (26.31)	
Unknown	133 (15.32)	4652 (6.16)	4785 (6.28)	
Injury severity				127.4 (<0.0001)
Died	297 (34.22)	22,013 (29.21)	22,310 (29.27)	
Injured	423 (48.73)	36,780 (48.81)	37,203 (48.81)	
Not injured	126 (14.52)	16,283 (21.61)	16,409 (21.53)	
Unknown	22 (2.53)	283 (0.37)	305 (0.40)	
License validity				398.2 (<0.0001)
Valid	504 (57.53)	62,305 (82.47)	62,809 (82.18)	
Invalid	313 (35.73)	11,909 (15.76)	12,222 (15.99)	
Unknown	59 (6.74)	1338 (1.77)	1397 (1.83)	
Drug or alcohol tests, tested only				1.5 (0.2270)
Tested, negative	0 (0.00)	93 (0.43)	93 (0.42)	
Tested, positive	340 (100.00)	21,661 (99.57)	22,001 (99.58)	
Race in death certificate, w/o N/A				4258.0 (<0.0001)
White, non-Hispanic	47 (15.82)	12,296 (55.85)	12,343 (55.32)	
Black, non-Hispanic	1 (0.34)	2636 (11.97)	2637 (11.82)	
Hispanic, White or Black	33 (11.11)	3692 (16.77)	3725 (16.70)	
Native American	192 (64.65)	431 (1.96)	623 (2.79)	
Asian or Pacific Islander	1 (0.34)	473 (2.15)	474 (2.12)	
Multiple/Other	0 (0.00)	238 (1.08)	238 (1.07)	
Unknown	23 (7.74)	2249 (10.22)	2272 (10.18)	
Previous moving violation combined				305.5 (<0.0001)
Yes	207 (23.63)	21,792 (28.84)	21,999 (28.78)	
No	548 (62.56)	51,342 (67.96)	51,890 (67.89)	
Unknown	121 (13.81)	2418 (3.20)	2539 (3.32)	
**Passenger Characteristics**	1667 (1.30)	126,080 (98.70)	127,747	
Passenger age (years)				8.3 (0.1405)
0 to 2	173 (10.38)	13,526 (10.73)	13,699 (10.72)	
3 to 8	323 (19.38)	26,735 (21.20)	27,058 (21.18)	
9 to 12	217 (13.02)	17,245 (13.68)	17,462 (13.67)	
13 to 14	176 (10.56)	11,383 (9.03)	11,559 (9.05)	
15 to 17	431 (25.85)	32,022 (25.40)	32,453 (25.40)	
18 to 19	347 (20.82)	25,169 (19.96)	25,516 (19.97)	
Passenger belt status				470.3 (<0.0001)
Restrained	551 (33.05)	74,764 (59.30)	75,315 (58.96)	
Unrestrained	919 (55.13)	42,892 (34.02)	43,811 (34.40)	
Unknown	197 (11.82)	8424 (6.68)	8621 (6.75)	
Injury severity				79.7 (<0.0001)
Died	383 (22.98)	27,869 (22.10)	28,252 (22.12)	
Injured	1006 (60.35)	69,296 (54.96)	70,302 (55.03)	
Not injured	264 (15.84)	28,679 (22.75)	28,943 (22.66)	
Unknown	14 (0.84)	236 (0.19)	250 (0.20)	
Seating position				531.2 (<0.0001)
Front	514 (30.83)	44,751 (35.49)	45,265 (35.43)	
Rear side	598 (35.87)	56,291 (44.65)	56,889 (44.53)	
Rear middle	193 (11.58)	14,948 (11.86)	15,141 (11.85)	
Other	94 (5.64)	4394 (3.49)	4488 (3.51)	
Unknown	268 (16.08)	5696 (4.52)	5964 (4.67)	
**Vehicle Characteristics**	876 (1.15)	75,552 (98.85)	76,428	
Vehicle model year				126.1 (<0.0001)
<1994	240 (27.40)	18,029 (23.86)	18,269 (23.90)	
1994–1997	189 (21.58)	16,435 (21.75)	16,624 (21.75)	
1998–2004	328 (37.44)	31,118 (41.19)	31,446 (41.14)	
2005–2008	91 (10.39)	7707 (10.20)	7798 (10.20)	
2009–2011	7 (0.80)	1427 (1.89)	1434 (1.88)	
2012–2014	8 (0.91)	741 (0.98)	749 (0.98)	
Unknown	13 (1.48)	95 (0.13)	108 (0.14)	
Model type				49.1 (<0.0001)
Passenger cars	386 (44.06)	39,025 (51.65)	39,411 (51.57)	
SUV	177 (20.21)	16,419 (21.73)	16,596 (21.71)	
Vans	88 (10.05)	7273 (9.63)	7361 (9.63)	
Pickups	225 (25.68)	12,835 (16.99)	13,060 (17.09)	
**Crash Characteristics**	876 (1.15)	75,552 (98.85)	76,428	
Day/night				22.4 (<0.0001)
Day	404 (46.12)	38,552 (51.03)	38,956 (50.97)	
Night	289 (32.99)	25,500 (33.75)	25,789 (33.74)	
Unknown	183 (20.89)	11,500 (15.22)	11,683 (15.29)	
Weekday/weekend				22.6 (<0.0001)
Weekday	483 (55.14)	43,991 (58.23)	44,474 (58.19)	
Weekend	389 (44.41)	31,513 (41.71)	31,902 (41.74)	
Unknown	4 (0.46)	48 (0.06)	52 (0.07)	
Rollover				318.2 (<0.0001)
Yes	474 (54.11)	20,458 (27.08)	20,932 (27.39)	
No	402 (45.89)	55,094 (72.92)	55,496 (72.61)	
Ejected				328.7 (<0.0001)
Yes	199 (22.93)	8377 (11.12)	8576 (11.25)	
No	635 (73.16)	66,627 (88.41)	67,262 (88.24)	
Unknown	34 (3.92)	355 (0.47)	389 (0.51)	
Rollover or ejection				269.9 (<0.0001)
Yes	502 (57.31)	24,278 (32.13)	24,780 (32.42)	
No	362 (41.32)	50,899 (67.37)	51,261 (67.07)	
Unknown	12 (1.37)	375 (0.50)	387 (0.51)	
Manner of collision				176.2 (<0.0001)
Non-collision	555 (63.36)	33,642 (44.53)	34,197 (44.14)	
Rear-end	47 (5.37)	5946 (7.87)	5993 (7.84)	
Head-on	116 (13.24)	10,263 (13.58)	10,379 (13.58)	
Angle	122 (13.93)	22,759 (30.12)	22,881 (29.94)	
Sideswipe	24 (2.74)	2561 (3.39)	2585 (3.38)	
Other	5 (0.57)	265 (0.35)	270 (0.35)	
Unknown	7 (0.80)	116 (0.15)	123 (0.16)	
**Roadway Characteristics**	876 (1.15)	75,552 (98.85)	76,428	
Number of lanes				65.9 (<0.0001)
Non-trafficway	0 (0.00)	76 (0.10)	76 (0.10)	
One	4 (0.46)	196 (0.26)	200 (0.26)	
Two	209 (23.86)	12,540 (16.60)	12,749 (16.68)	
Three	3 (0.34)	1613 (2.13)	1616 (2.11)	
Four	7 (0.80)	2467 (3.27)	2474 (3.24)	
Five	2 (0.23)	481 (0.64)	483 (0.63)	
Six or more	0 (0.00)	353 (0.47)	353 (0.46)	
Unknown	651 (74.32)	57,826 (76.54)	58,477 (76.51)	
Trafficway (one/two-way)				45.0 (<0.0001)
Non-trafficway	0 (0.00)	76 (0.10)	76 (0.10)	
One-way	3 (0.34)	187 (0.25)	190 (0.25)	
Two-way, divided	36 (4.11)	6118 (8.10)	6154 (8.05)	
Two-way, not divided	186 (21.23)	11,190 (14.81)	11,376 (14.88)	
Other	0 (0.00)	187 (0.25)	187 (0.24)	
Unknown	651 (74.32)	57,794 (76.50)	58,445 (76.47)	
Traffic devices and signs				40.8 (<0.0001)
No controls	210 (23.97)	13,728 (18.17)	13,938 (18.27)	
Traffic signals	0 (0.00)	1855 (2.46)	1855 (2.43)	
Regulatory signs	16 (1.83)	2049 (2.71)	2065 (2.70)	
Other	2 (0.23)	145 (0.19)	147 (0.19)	
Unknown	648 (73.97)	57,775 (76.47)	58,423 (76.44)	
Route signing				417.8 (<0.0001)
Interstate	59 (7.13)	10,929 (14.53)	10,988 (14.45)	
Highway	416 (50.30)	30,547 (40.62)	30,963 (40.72)	
Country road	180 (21.77)	14,976 (19.91)	15,156 (19.93)	
Local street	29 (3.51)	15,102 (20.08)	15,131 (19.90)	
Other	143 (17.29)	3650 (4.85)	3793 (4.99)	
Traffic control device functioning				35.4 (<0.0001)
Not functioning properly	0 (0.00)	20 (0.03)	20 (0.03)	
Functioning properly	17 (1.94)	3999 (5.29)	4016 (5.25)	
No controls	210 (23.97)	13,728 (18.17)	13,938 (18.24)	
Unknown	649 (74.09)	57,805 (76.51)	58,454 (76.48)	

* Percentages may not add up to 100% due to rounding.

**Table 2:** Adjusted ORs (95% CIs) for infant, child and teen being restrained in the states with federally recognized Indian lands, FARS 2000–2014, categorized by passenger ages 0–12 and 13–19 years.

Variables	0–12 Adjusted, Restrained	13–19 Adjusted, Restrained
	*n* = 44,459	*n* = 50,565
Driver Characteristics		
Driver age (years)		
<20	Ref	Ref
20 to 44	2.023 (1.798, 2.276)	1.272 (1.208, 1.340)
45 to 64	1.840 (1.601, 2.115)	1.840 (1.687, 2.008)
≥65	2.234 (1.780, 2.803)	2.063 (1.667, 2.554)
Driver restraint use		
Not restrained	Ref	Ref
Restrained	7.759 (7.228, 8.330)	10.607 (10.046, 11.200)
Driver gender		
Male	Ref	Ref
Female	1.021 (0.958, 1.088)	1.074 (1.019, 1.132)
License validity		
Invalid	Ref	Ref
Valid	2.098 (1.930, 2.281)	1.500 (1.409, 1.598)
Drug or alcohol tests		
Tested, negative	Ref	Ref
Tested, negative	1.464 (0.609, 3.520)	0.870 (0.487, 1.555)
Not tested	1.699 (0.707, 4.084)	1.087 (0.608, 1.943)
Vehicle Characteristics		
Model type		
Passenger cars	Ref	Ref
SUV	0.820 (0.758, 0.887)	0.742 (0.696, 0.790)
Vans	0.695 (0.631, 0.766)	0.622 (0.566, 0.683)
Pickups	0.777 (0.705, 0.855)	0.754 (0.704, 0.807)
Crash Characteristics		
Indian land status of the crash site		
Non-Indian land	Ref	Ref
Indian land	0.535 (0.416, 0.715)	0.635 (0.500, 0.807)

**Table 3:** Unadjusted and adjusted ORs (95% CIs) for infant, child and teen restraint status on Indian lands in the states with federally recognized Indian lands, FARS 2000–2014, divided by passenger ages 0–12 and 13–19 years.

Variables	0–12 Unadjusted, Restrained	0–12 Adjusted, Restrained	13–19 Unadjusted, Restrained	13–19 Adjusted, Restrained
	*n* = 713	*n* = 520	*n* = 954	*n* = 628
Driver Characteristics				
Driver age (years)				
<20	Ref	Ref	Ref	Ref
20 to 44	3.726 (1.624, 8.549)	2.396 (0.826, 6.951)	1.618 (1.039, 2.519)	1.273 (0.716, 2.265)
45 to 64	4.895 (1.740, 13.775)	1.225 (0.324, 4.629)	12.090 (5.091, 28.709)	4.763 (1.464, 15.495)
≥65	1.899 (0.184, 19.636)	0.286 (0.012, 6.644)	10.558 (1.280, 87.603)	1.904 (0.040, 91.206)
Driver restraint use				
Not restrained	Ref	Ref	Ref	Ref
Restrained	8.496 (4.989, 14.468)	6.548 (3.262, 13.143)	22.887 (14.029, 37.336)	13.885 (7.702, 25.033)
Driver gender				
Male	Ref	Ref	Ref	Ref
Female	0.913 (0.556, 1.500)	0.711 (0.382, 1.324)	1.693 (1.109, 2.586)	1.300 (0.742, 2.278)
License validity				
Invalid	Ref	Ref	Ref	Ref
Valid	5.894 (3.314, 10.484)	2.662 (1.249, 5.674)	3.929 (2.512, 6.146)	1.738 (0.966, 3.129)
Drug or alcohol tests				
Tested, positive ^1^	Ref	Ref	Ref	Ref
Tested, negative	-	-	-	-
Not tested	1.577 (0.936, 2.658)	1.226 (0.646, 2.325)	4.426 (2.808, 6.977)	1.563 (0.827, 2.956)
Vehicle Characteristics				
Model type				
Passenger cars	Ref	Ref	Ref	Ref
SUVs	0.746 (0.395, 1.411)	0.937 (0.422, 2.081)	1.300 (0.755, 2.238)	0.489 (0.229, 1.046)
Vans	1.023 (0.475, 2.204)	1.103 (0.425, 2.862)	2.672 (1.232, 5.795)	0.932 (0.344, 2.523)
Pickups	0.532 (0.279, 1.014)	0.553 (0.246, 1.244)	0.966 (0.581, 1.606)	0.604 (0.307, 1.185)

^1^ Positive test result was made the reference value here because no driver was tested negative for drugs or alcohols on Indian lands.

**Table 4:** Unadjusted and adjusted ORs (95% CIs) for infant, child and teen restraint status on non-Indian lands in the states with federally recognized Indian lands, FARS 2000–2014, categorized by passenger ages 0–12 and 13–19 years.

Variables	0–12 Unadjusted, Restrained	0–12 Adjusted, Restrained	13–19 Unadjusted, Restrained	13–19 Adjusted, Restrained
	*n* = 57,506	*n* = 43,939	*n* = 68,574	*n* = 49,937
Driver Characteristics				
Driver age (years)				
<20	Ref	Ref	Ref	Ref
20 to 44	2.276 (2.063, 2.511)	2.013 (1.787, 2.267)	1.228 (1.179, 1.280)	1.272 (1.208, 1.339)
45 to 64	2.367 (2.110, 2.657)	1.842 (1.601, 2.119)	2.701 (2.518, 2.898)	1.829 (1.676, 1.996)
≥65	3.050 (2.524, 3.686)	2.249 (1.790, 2.825)	3.157 (2.649, 3.763)	2.066 (1.669, 2.558)
Driver restraint use				
Not restrained	Ref	Ref	Ref	Ref
Restrained	8.509 (8.002, 9.049)	7.799 (7.261, 8.378)	12.459 (11.879, 13.068)	10.565 (10.004, 11.159)
Driver gender				
Male	Ref	Ref	Ref	Ref
Female	1.170 (1.111, 1.233)	1.026 (0.962, 1.093)	1.593 (1.527, 1.661)	1.074 (1.018, 1.132)
License validity				
Invalid	Ref	Ref	Ref	Ref
Valid	2.608 (2.431, 2.798)	2.097 (1.928, 2.281)	2.036 (1.935, 2.143)	1.495 (1.403, 1.592)
Drug or alcohol tests				
Tested, negative	Ref	Ref	Ref	Ref
Tested, positive	1.219 (0.559, 2.658)	1.462 (0.608, 3.514)	0.985 (0.592, 1.639)	0.861 (0.482, 1.535)
Not tested	2.142 (0.983, 4.667)	1.695 (0.706, 4.073)	2.014 (1.210, 3.350)	1.074 (0.602, 1.916)
Vehicle Characteristics				
Model type				
Passenger cars	Ref	Ref	Ref	Ref
SUVs	0.938 (0.878, 1.003)	0.819 (0.757, 0.887)	0.934 (0.887, 0.984)	0.744 (0.698, 0.793)
Vans	0.968 (0.892, 1.050)	0.691 (0.627, 0.762)	1.128 (1.046, 1.217)	0.619 (0.563, 0.680)
Pickups	0.766 (0.708, 0.830)	0.784 (0.711, 0.864)	0.700 (0.663, 0.739)	0.755 (0.705, 0.808)
